# Management of complicated primary intestinal lymphoma in the emergency setting. Experience at quaternary hospital

**DOI:** 10.1007/s00423-025-03821-6

**Published:** 2025-07-24

**Authors:** Manuel José Torres-Jurado, Jaime López-Sánchez, Andrés E. Valera-Montiel, Laura Vicente-González, Jacobo Trébol-López, José Edecio Quiñones-Sampedro, Francisco Blanco-Antona

**Affiliations:** 1 General and Digestive Surgery Department, University Hospital of Salamanca, Salamanca, Spain; 2 Biomedical Research Institute of Salamanca (ISBSAL), Salamanca, Spain; 3 University of Salamanca, Salamanca, Spain

**Keywords:** Primary small bowel lymphoma, B-cell lymphoma, T-cell lymphoma, Postoperative complications, Survival, Emergency surgery

## Abstract

**Purpose:**

Until now, there have been almost no published references that focus on the postoperative results of patients with complicated primary small bowel lymphoma (PSBL). Our goal is to analyze the postoperative results of patients who underwent emergency surgery for PSBL.

**Methods:**

Retrospective observational study of patients diagnosed with complicated PSBL who underwent emergency surgery between 2016 and 2024. The presented gastrointestinal symptoms or findings in the imaging tests that showed hemorrhage, obstruction, or perforation. The incidence of postoperative complications, as well as total (TS) and disease-free survival (DFS) after surgery, were evaluated.

**Results:**

Seventeen patients with the diagnosis of PSBL were analyzed. The most common clinical manifestation was intestinal obstruction (41.2%). The most frequent location was the ileum (47%). 40% of patients were diagnosed with diffuse large B-cell lymphoma (41.2%). In total, 12 patients (70.7%) had Lugano stage I disease. The mean TS of the patients was 39 months (range: 32–46). The TS of the patients with B-cell lymphoma was higher than T-cell lymphoma (B-cell lymphoma 27 months vs. T-cell lymphoma 8 months, *P* = 0.018). The median DFS was 41 months (range: 33–49). The DFS of patients with B-cell lymphoma was higher than T-cell lymphoma (27 months vs. 6 months. *P* = 0.019).

**Conclusions:**

Emergency surgery due to complications in patients with primary intestinal lymphoma does not have negative predictive value on their survival. The prognosis is worse in patients with T-cell primary intestinal lymphomas with a higher rate of postoperative complications and recurrence.

## Introduction

Tumors of the small bowel represent less than 5% of all gastrointestinal neoplasms, with primary small bowel lymphomas (PSBL) accounting for 10–15% of these cases. Histologically, the majority of PSBL are classified as non-Hodgkin lymphomas (NHL) [1–5], with the most prevalent subtypes including diffuse large B-cell lymphoma, B-cell mucosa-associated lymphoid tissue (MALT) lymphoma of the extranodal marginal zone, and mantle cell lymphoma. Intestinal T-cell lymphoma is less common, often associated with celiac disease, and primarily localized to the jejunoileal region [3, 6, 7].

PSBL is frequently diagnosed at advanced stages due to complications such as bowel obstruction, perforation, or hemorrhage [3, 4]. These complications often necessitate urgent surgical intervention, with the main goal being to address the life-threatening issues associated with bowel obstruction or perforation, while also considering the oncological implications. The management of PSBL in an emergency setting remains challenging due to the multifactorial nature of the disease and its late-stage presentation. Urgent surgical procedures, including bowel resection or stoma formation, are commonly performed to stabilize the patient. However, it is essential that these interventions are carefully balanced with the overall treatment strategy for lymphoma, which typically includes chemotherapy, immunotherapy, and sometimes radiotherapy, depending on the subtype and stage of the disease.

In critical situations, the role of surgery extends beyond symptoms management, as it can influence the subsequent course of treatment and overall prognosis. While surgery may be essential to manage complications, its timing and extent are crucial to ensuring the feasibility of postoperative oncological therapies, particularly chemotherapy. In cases of extensive bowel involvement, more aggressive surgical approach may be required. However, decisions regarding such interventions must take account prognostic indicators such as the International Prognostic Index (IPI) and Dawson’s classification [1, 8]. The IPI, which incorporates factors such as age, disease stage, performance status, and serum markers, is instrumental in predicting long-term survival. Dawson’s classification, which evaluates the extent of nodal involvement, also aids in determining the appropriateness of surgical versus palliative approaches [1].

While elective surgery for PSBL is tipically planned with a focus on improving long-term outcomes, emergency surgeries are reactive and driven by acute clinical presentations [2]. There is ongoing debate regarding whether early surgical management in such cases influences disease progression or alters survival outcomes [1]. Specifically, concerns have been raised about the potential impact of surgical complications and efficacy of post-operative chemotherapy regimens [6]. The limited number of studies examining the management of PSBL with complications often involve heterogeneous patient populations, complicating the establishment of standardized treatment guidelines [4, 5]. This underscores the need for further research to develop evidence-based protocols for managing these complex cases effectively.

This study aims to analyze the comprehensive management of patients undergoing emergency surgery for PSBL, with a specific focus on the integration of early surgical intervention with subsequent oncological treatments.

## Materials and methods

### Design

This is a retrospective observational study on a prospective database including all the patients between June 2016 and February 2024 diagnosed with PSBL at the time of emergency surgery due to tumor-related complications; none had a prior diagnosis of PSBL before presenting with acute symptoms necessitating surgical intervention. These cases were managed in the Department of General Surgery of the University Hospital of Salamanca. Our hospital is a fourth level care center and a national reference center for the management and treatment of highly complex malignant hemopathies. The research protocol for this observational, retrospective, and comparative study followed the Declaration of Helsinki’s ethical requirements. This study followed the STROBE guidelines for reporting observational studies [9].

### Study objectives and outcomes

The primary outcome of this study was to evaluate the overall survival (OS) of patients undergoing emergency surgery for primary small bowel lymphoma (PSBL) [1–8]. Secondary outcomes included disease-free survival (DFS), postoperative complication rates classified according to the Clavien-Dindo classification [10] and the Comprehensive Complication Index (CoCI), and the response to adjuvant therapy [4, 6].

### Study population

The study included patients who presented gastrointestinal symptoms or findings in the imaging tests that showed digestive hemorrhage, obstruction, or perforation not derived from endoscopic treatment, and who required emergency surgery. All cases were confirmed as PSBL based on histopathological examination following surgery. These patients were managed in the Department of General Surgery of the University Hospital of Salamanca.

Inclusion criteria: Patients diagnosed with primary small bowel lymphoma (PSBL) who presented with acute gastrointestinal complications such as hemorrhage, obstruction, or perforation, and who required emergency surgery in the Department of General Surgery of the University Hospital of Salamanca between June 2016 and February 2024. Diagnosis was confirmed postoperatively by histopathological.

Exclusion criteria: Patients with secondary involvement of the small bowel by lymphoma originating from other primary sites were excluded. Additionally, patients whose complications were not due to primary intestinal lymphomas, despite initial suspicion, as well as those undergoing elective surgery, were excluded.

### Variables and definitions

The following variables were analyzed: demographic information, comorbidity according to the Charlson Comorbidity Index (ChCI), clinical presentation of the disease, preoperative analytical data, histological type, tumor stage, type of surgical approach, surgical procedure, postoperative complications according to the Clavien-Dindo classification [10] and the Comprehensive Complication Index (CoCI), postoperative chemotherapy, total survival (TS) and disease-free survival (DFS). The diagnosis was established according to the anatomopathological study of the surgical sample, which is based on the 2016 review of the World Health Organization (WHO) classification [11]. The staging was conducted according to the Lugano classification [12]. The age-adjusted International Prognostic Index (IPI) for lymphomas was used as a predictor of prognosis [13]. Dawson’s criteria were used to define PSBL with locoregional involvement [1].

Emergency surgery was indicated for PSBL patients presenting acute complications such as intestinal obstruction, perforation, or active bleeding, confirmed by clinical and radiological assessment (primarily CT scans). Surgery was decided based on the severity of symptoms, imaging evidence of bowel compromise (e.g., free air, significant distension, ongoing hemorrhage), and the patient’s overall clinical status. Urgent intervention was prioritized for patients showing systemic deterioration or signs of peritonitis to manage life-threatening complications and stabilize them before further oncological treatment.

### Statistical analysis

The Shapiro—Wilks test was used to study the distribution of variables. The analysis of the relationship between variables was conducted with Spearman’s correlation for quantitative variables and the chi-squared test for categorical variables. For the combination of both categories, the Wilcoxon test and the Kruskal—Wallis test were used.

The survival study used Kaplan—Meier estimators and the log-rank test to compare different groups. Statistical significance was established for *p* < 0.05. The statistical study was conducted with the R statistical software and the survival [14] and survminer packages [15] (V.4.1.2+, R Core Team, Vienna, https://www.R-project.org/).

## Results

### Clinical and pathological characteristics

Seventeen patients with the diagnosis of PSBL were analyzed during the study period (13 men/4 women). The median age was 73 years (range: 46–81 years) (Table 1). The median Body Mass Index (BMI) was 23.4 kg/m^2^ (range: 21.8–24.9 kg/m^2^). Most of the patients (53%) presented a score less than 2 points in the ChCI. The most common clinical manifestation was intestinal obstruction (41.2%) followed by perforation (23.5%), simultaneous obstruction and perforation (11.8%), simultaneous obstruction and hemorrhage (11.8%) and simultaneous intestinal perforation with hemorrhage (5.9%). The most frequent location was the ileum (47%), followed by the jejunum (41.2%) and the ileocecal region (11.8%). 40% of patients were diagnosed with diffuse large B-cell lymphoma (41.2%) followed by T-cell lymphoma (35.3%), follicular lymphoma (11.8%), marginal zone B-cell lymphoma (5.9%) and plasmablastic lymphoma (5.9%) (Table 2). All the patients met Dawson’s criteria. There was only one case of a multiple gastrointestinal lymphoma with 2 foci in the small intestine and 1 in the cecum. In total, 12 patients (70.6%) had Lugano stage I disease, and 5 (29.4%) had Lugano stage II disease.

**Table 1:** Characteristics of the patients

Variable	*N* = 17
Age	73 (56. 81)
Sex	
Men	13 (76.5%)
Women	4 (23.5%)
Body mass index (BMI)	24.28 (21.80, 24.90)
Arterial hypertension (AHT)	8 (47%)
Dyslipidemia	8 (47%)
Diabetes mellitus (DM)	5 (29.4%)
Cardiovascular disease	6 (35.2%)
Cerebrovascular disease	0 (0%)
Chronic kidney disease	1 (5.9%)
Respiratory disease	2 (11.7%)
Previous oncological disease	5 (29.4%)
Charlson Comorbidity Index (CCI)	
Ausencia de comorbilidad (0–1 punto)	7 (41.2%)
Comorbilidad baja (2 puntos)	1 (5.9%)
Comorbilidad alta (> 3 puntos)	9 (53%)
International Prognostic Index (IPI)	
Low risk	8 (47%)
Middle-low risk	5 (29.4%)
Middle-high risk	1 (5.9%)
High risk	3 (17.6%)
Celiac disease	2 (11.7%)
Clinical presentation	
Intestinal perforation	4 (23.5%)
Digestive hemorrhage	1 (5.9%)
Intestinal obstruction	7 (41.2%)
Intestinal obstruction and hemorrhage	2 (11.8%)
Intestinal obstruction and perforation	2 (11.8%)
Intestinal perforation and hemorrhage	1 (5.9%)
Location	
Duodenum	0 (0%)
Jejunum	7 (41.2%)
Ileum	8 (47%)
Ileocolic region	2 (11.8%)

**Table 2:** Histology and staging of the patients

Variable	*N* = 17
Lugano Staging System	
I	12 (70.6%)
II	5 (29.4%)
II Bulky	0 (0%)
III	0 (0%)
IV	0 (0%)
Mesenteric infiltration	7 (41.2%)
Bone marrow biopsy	
Not conducted	11 (64.7%)
Normal	6 (35.3%)
Deauville criteria	
1	13 (76.5%)
2	1 (5.9%)
3	0 (0%)
4	0 (0%)
5	3 (17.6%)
Anatomopathological analysis	
B-cell lymphoma	
Large B-cell lymphoma	7 (41.2%)
Follicular lymphoma	2 (11.8%)
Marginal zone lymphoma	1 (5.9%)
Plasmablastic lymphoma	1 (5.9%)
T-cell lymphoma	6 (35.3%)

### Surgery

**Fig. 1:**
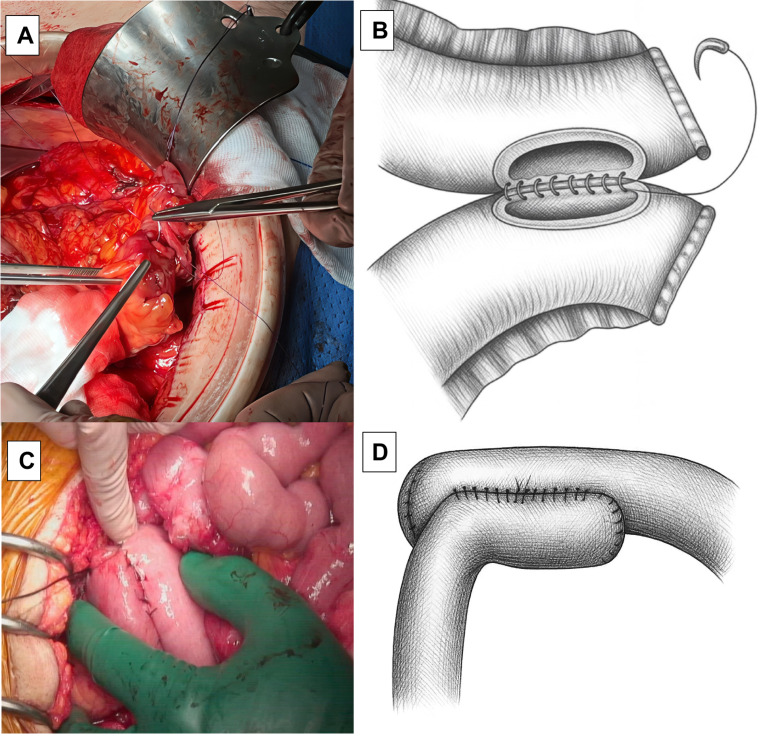
Intraoperative view of a manual two-layer isoperistaltic ileocolic anastomosis following ileocolic resection for primary intestinal lymphoma. The anastomosis was performed with anterior and posterior reinforcing sutures to ensure optimal sealing and reduce the risk of leakage.**A** Intraoperative image showing the ileocolic anastomosis.**B** Corresponding schematic illustration depicting the placement of the posterior layer sutures.**C** Intraoperative image of a manual two-layer ileoileal anastomosis.**D** Corresponding schematic illustration showing the anterior reinforcing sutures placed with silk

All the emergency surgical procedures are summarized in Table 2. The most frequent approach was open surgery (88.2%) and two cases (11.8%) underwent laparoscopic surgery. 94.1% of the patients required intestinal resection with a median length of the resected bowel of 20 cm (range: 15–30 cm), and in two cases a right-sided colon resection was necessary due to the adjacent spread of the disease. One patient underwent primary closure of the perforation after wound excision and edge biopsy. An R0 excision—defined as a complete tumor resection with no microscopic residual disease—was performed in 14 cases (10 cases of B-cell lymphoma and 4 cases of T-cell lymphoma), and an R2 resection—defined as a resection with macroscopic residual tumor—was achieved in 3 cases (2 cases of T-cell lymphoma and 1 case of B-cell lymphoma). In all cases, regional lymphadenectomy was performed as part of the oncologic resection, primarily for staging and diagnostic purposes. A systematic extended lymphadenectomy was not routinely performed due to the emergency nature of the procedures and the hemodynamic condition of the patients. The median number of lymph nodes resected was [10] (range: 5–14), and histopathological involvement was confirmed in 17.6% of the cases. An antiperistaltic latero-lateral mechanical anastomosis was the most common approach (47%), followed by isoperistaltic latero-lateral manual approach (35.3%) (Fig. 1A and D), and antiperistaltic latero-lateral manual approach (5.9%). Only one patient required a doble terminal jejunostomy (5.9%).

Emergency surgeries commonly revealed intestinal perforation, tumor-related obstruction, or severe bowel wall inflammation. Some cases showed intestinal necrosis and localized or diffuse peritonitis. Tumor extent and regional lymph node involvement were assessed intraoperatively to guide the type of surgery performed, such as segmental resection, bypass, or decompression, and to plan postoperative and oncological management.

### Postoperative results

The postoperative complications according to the Clavien-Dindo classification are shown in the Table 3. Most of the complications were minor (76.5%), with predominance of postoperative paralytic ileus (45%). The rate of major complications (Clavien-Dindo > 3) was 23.6%. Re-interventions within the first 30 days was needed in 11.9%, including one death after multiple postoperative complications. The median hospital stay was 10 days (range: 4–45 days) (Table 4).

**Table 3:** Surgical treatment and results

Variable	*N* = 17
Approach	
Open surgery	15 (88.2%)
Laparoscopic surgery	2 (11.8%)
Primary closure of the perforation	1 (5.9%)
Intestinal resection	16 (94.1%)
Length of the intestinal resection (cm)	20 (15, 30)
Ileocolic resection	2 (13.3%)
Type of anastomosis	
Not conducted	2 (11.8%)
Antiperistaltic latero-lateral mechanical	8 (47%)
Antiperistaltic latero-lateral manual	1 (5.9%)
Isoperistaltic latero-lateral manual	6 (35.3%)
Stoma	
Without stoma	16 (94.1%)
Terminal ileostomy	0 (0%)
Double terminal jejunostomy	1 (5.9%)
Re-intervention for complications	2 (11.8%)
Re-intervention for local recurrence	1 (5.9%)
Postoperative complications (Clavien-Dindo)	
Grade I	11 (64.7%)
Grade II	2 (11.8%)
Grade IIIa	1 (5.9%)
Grade IIIb	2 (11.8%)
Grade IVa	0 (0%)
Grade V	1 (5.9%)
Estancia hospitalaria (días)	10 (4, 45)
Adjuvant chemotherapy (CT)	10 (58.8%)
Response to adjuvant CT	
Yes	7(70%)
No	3(30%)
Complete remission	8(47%)
Number of days	30 (0, 253)
Number of cycles	6 (4, 6)

**Table 4:** Analysis of B and T lymphoma subgroups

	LINFOMA B, *N* = 11^1^	LINFOMA T, *N* = 6^1^	*p*-value^2^
International Prognostic Index (IPI)			0.8
Low risk	5 (45.4%)	3 (50%)	
Middle-low risk	4 (36.4%)	1 (16.6%)	
Middle-high risk	0 (0%)	1 (16.6%)	
High risk	2 (18.2%)	1 (16.6%)	
Clinical presentation			0.5
Intestinal perforation	2 (18.2%)	2 (33.3%)	
Digestive hemorrhage	0 (0%)	1 (16.7%)	
Intestinal obstruction	4 (36.4%)	3 (50%)	
Intestinal obstruction and hemorrhage	2 (18.2%)	0 (0%)	
Intestinal obstruction and perforation	2 (18.2%)	0 (0%)	
Intestinal perforation and hemorrhage	1 (9.1%)	0 (0%)	
Location			< 0.001
Jejunum	0 (0%)	6 (100%)	
Ileum	11 (100%)	0 (0%)	
Approach			0.5
Open surgery	9 (81.8%)	6 (100%)	
Laparoscopic surgery	2 (18.2%)	0 (0%)	
Primary closure of the perforation	0 (0%)	1 (16.7%)	0.4
Intestinal resection	11 (100%)	5 (83.3%)	
Length of the intestinal resection (cm)	20 (13, 25)	23 (15, 40)	0.8
Oncological resection			0.5
R0	10 (90.9%)	4 (66.7%)	
R1	0 (0%)	0 (0%)	
R2	1 (9.1%)	2 (33.3%)	
Number of lymph nodes resected	9 (4,10)	12 (7,14)	0.3
Type of anastomosis			0.2
Not conducted	0 (0%)	2 (33.3%)	
Antiperistaltic latero-lateral mechanical	5 (45.4%)	3 (50%)	
Antiperistaltic latero-lateral manual	1 (9.1%)	0 (0%)	
Isoperistaltic latero-lateral manual	5 (45.4%)	1 (16.7%)	
Stoma			0.4
Without stoma	11 (100%)	5 (83.3%)	
Double terminal jejunostomy	0 (0%)	1 (16.7%)	
Reintervention	2 (18.2%)	1 (16.7%)	> 0.9
Postoperative complications (Clavien-Dindo)			> 0.9
Grade I	7 (63.6%)	4 (66.7%)	
Grade II	1 (9.1%)	1 (16.7%)	
Grade IIIa	1 (9.1%)	0 (0%)	
Grade IIIb	1 (9.1%)	1 (16.7%)	
Grade IVa	0 (0%)	0(0%)	
Grade IVb	0 (0%)	0 (0%)	
Grade V	1 (9.1%)	0 (0%)	
Comprehensive complication index (CoCI)	9 (9, 28)	16 (9, 35)	0.6
Hospital stay (days)	8 (7, 18)	11 (9, 12)	0.4
Total survival (TS) (days)	800 (526, 1,062)	226 (120, 720)	0.018
Disease-free survival (DFS) (days)	798 (340, 1,012)	136 (11, 234)	0.019
Complete remission (days)	59 (0, 519)	0 (0, 63)	0.4

^1^n (%); Median (Q1, Q3)

^2^Fisher’s exact test; Wilcoxon rank sum exact test; Wilcoxon rank sum test

### Adjuvant therapy

**Fig. 2:**
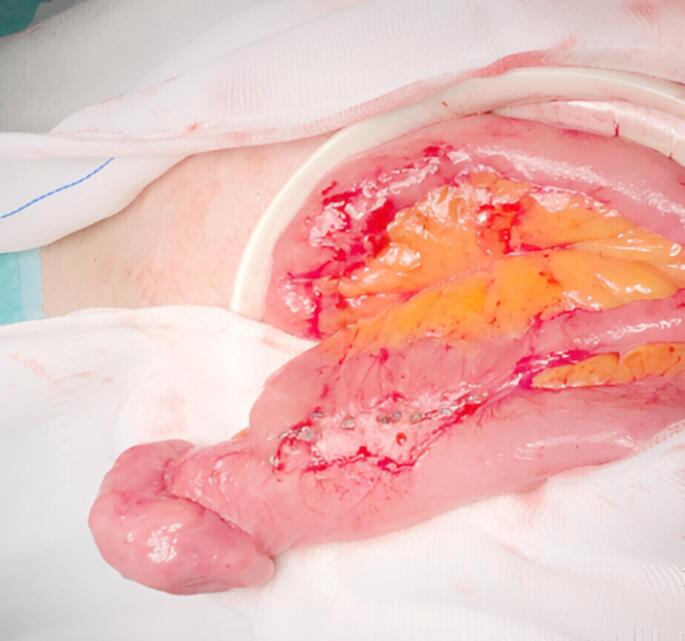
Intraoperative image of a re-intervention due to local recurrence in the anastomosis of a plasmablastic lymphoma

Ten patients (58.8%) received adjuvant therapy, with an initial response rate of 70% (7/10). Complete remission after chemotherapy was achieved in 47% of the cases. One case of local recurrence in the anastomosis required re-intervention in a patient without, postoperative adjuvant therapy (Fig. 2).

### Survival

**Fig. 3:**
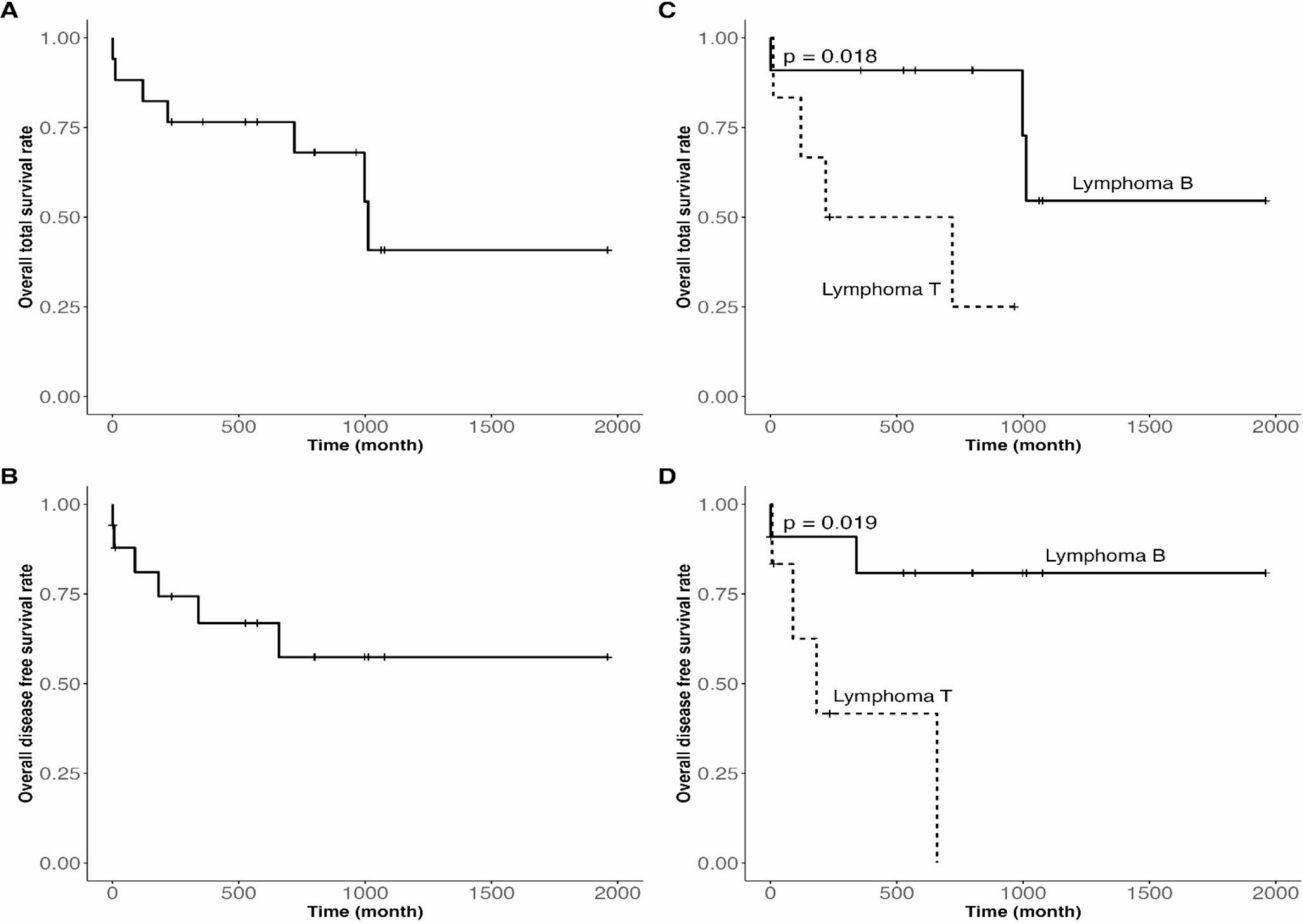
Kaplan-Meier curves for total survival (**A**) and disease-free survival (**B**) in the sample. Analysis of subgroups based on the anatomopathological results with log-rank test (**C**, **D**)

The mean TS of the patients was 39 months (range: 32–46) (Fig. 3A). The TS of the patients with B-cell lymphoma was higher than T-cell lymphoma (B-cell lymphoma 27 months vs. T-cell lymphoma 8 months, *p* = 0.018) (Fig. 3B). The median DFS was 41 months (range: 33–49) (Fig. 3C). The DFS of patients with B-cell lymphoma was higher than T-cell lymphoma (27 months vs. 6 months. *p* = 0.019) (Fig. 3D).

During the follow-up, 6 deaths were registered, related to systemic progression of the disease. One patient died due to hemophagocytic syndrome secondary to lymphoma progression. There was one case of death caused by septic shock after one re-intervention due to anastomosic leak. Another patient died in the inmediate postoperative period due to an anoxic encephalopathy secondary to multiple organ failure. Two patients died from respiratory infections due to immunosuppression from adjuvant treatment. The last death registered was secondary to squamous cell carcinoma of the auricle in an immunosuppressed patient several months after the surgery.

## Discussion

We present the results of the management series of patients with complicated PSBL who required emergency surgery without a preoperative diagnosis, and an analysis based on the histological type of the tumor.

PSBL are rare and there are few available data to guide treatment [4, 16, 17]. Surgery is rarely indicated because intestinal involvement is generally diffuse, and systemic chemotherapy is the standard treatment [3, 4].

Due to their low incidence and their histological heterogeneity, there are few publications on this condition, with small sample sizes and single-center studies which include all types of gastrointestinal lymphomas [18, 19]. Therefore, there is no clear evidence on the therapeutic management of this condition. There is a tendency to use mainly chemo and radiotherapy, and to limit the role of surgery to diagnostic purposes, to solve complications or to treat cases of refractory and localized disease [18–22].

The median age in our series, as well as the predominance of male patients (76.5%) were similar to the data observed in other studies published to date [18, 22, 23]. We have reported a wide variety of histological subtypes for primary lymphoma, with large B-cell lymphoma as the most common, in line with previous studies [1–4]. The ileocecal region was the most common location, according to the data reported by Lida et al. [4]. The most common clinical presentation in our series was intestinal obstruction, observed at a higher proportion than reported in other studies, such as Hong et al., who described 37% of cases presenting with obstruction, and Kim et al., who reported 16.5% [22, 23]. This difference could be attributed to the fact that a considerable number of patients in those studies underwent elective surgery. Notably, our findings contrast with more recent literature where primary small bowel lymphoma (PSBL) is more frequently associated with perforation as the initial presentation [24, 25]. This discrepancy may reflect variations in patient populations, disease stage at presentation, or timing of diagnosis. Radiological evaluation, particularly computed tomography (CT) scans, was fundamental in assessing complications such as obstruction, perforation, or hemorrhage. CT imaging findings informed the urgency and nature of surgical intervention, facilitating prompt and appropriate management in emergency settings. Early identification of radiological signs indicating bowel compromise is crucial to optimize patient outcomes and guide surgical decision-making. 70.6% of the patients in our study presented Lugano stage I disease, and 82.3% had an IPI score ≤ 2. The prognostic staging of the disease in our patients was similar to the series published in the literature [23].

**Fig. 4:**
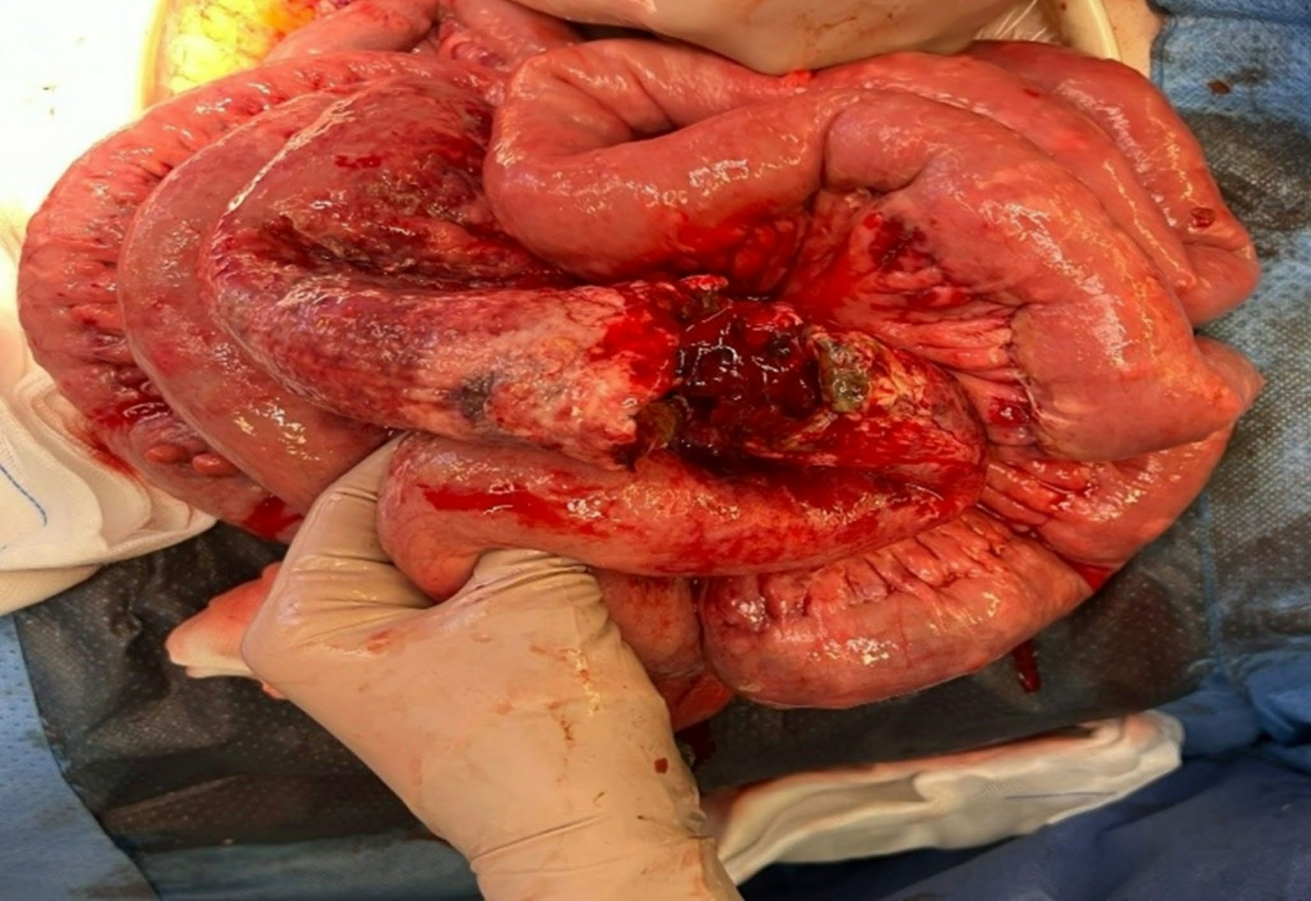
Intraoperative image of T-lymphoma in the jejunum with perforation and a thickened 30-cm segment with associated peritoneal implants

Primary anastomosis was performed in 88.2% of the patients in our cohort. This variable was not registered in the more recent publications, because most patients underwent elective surgery. Among the few studies which do specify the type of surgery (emergency/elective), Hong et al.. only report 15 cases (18.2%) of emergency surgeries and do not specify whether stoma was needed or not [23]. In our series, anastomosis could not be performed in one patient due to severe hemodynamic instability, and because the patient had the highest tumor load and presented a very widespread peritoneal lymphomatosis, which required a double jejunostomy (Fig. 4).

Regarding the postoperative complications, one patient required re-intervention due to anastomotic leak, and he underwent ileostomy, followed by admission to the ICU, where he died due to multiple organ failure. Another patient underwent a re-intervention due to the suspicion of an anastomotic leak, which was not confirmed during surgery. The same patient was later discharged without further complications. One patient required surgical re-intervention due to evisceration following laparotomy, and a new mesh reinforcement was used. Our rate of major complications was similar to the values reported in other series. However, the percentage of premature deaths related to these complications was lower in our sample (5.9% vs. 42.8%) than the data reported by *Hong et al.* [22]. Other minor postoperative complications included intraabdominal collection with percutaneous drainage and postoperative anemia which required transfusion. Another patient died in the postoperative stage due to cardiopulmonary arrest.

Only a few studies have reported survival outcomes following emergency surgery specifically for complications arising from primary small bowel tumors, rather than from general causes of abdominal emergencies such as perforated diverticulitis, perforated peptic ulcer disease, or adhesive small bowel obstruction. Among primary tumors of the small intestine, lymphoma—particularly T-cell lymphoma—has been reported to carry the highest risk of spontaneous perforation [24]. In our study, the general TS rate after one year was 76.5%, with 90,9% in the cases of B-cell lymphoma and 50% in the cases of T-cell lymphoma. These total survival rates may be explained by the tumor stage of our patients, because 47% had a low-risk lymphoma (IPI 0–1 points), and 70.6% had a stage I disease (Lugano Staging System). This factor could reinforce our survival results because our sample had a very similar basal situation (stage and IPI), as has been mentioned above. The early stages and International Prognostic Index (IPI) are predictors for higher survival rates. In the case of IPI, its prognostic relevance is applicable to cases of aggressive NHL, but not to patients with indolent lymphomas [14]. There is a notable difference in overall survival (OS) rates between the studies by Kim et al. [23] and Hong et al. [22]. Kim et al. reported on 162 patients with small bowel non-Hodgkin lymphoma, of whom 74 (45.7%) underwent surgery—both elective and emergency—with a 5-year OS rate of 77% for those who had surgery compared to 57% for those treated non-surgically. Conversely, Hong et al. included 82 patients with intestinal lymphoma, 62 (75.6%) of whom underwent surgery; among these, 38 (61.3%) underwent surgery due to tumor-related complications, resulting in a 5-year OS of only 26.7% for the emergency surgery group. These two studies are not directly comparable, as neither specifically compares 5-year OS rates exclusively for patients requiring emergency surgery for primary small bowel tumors. In our current study, we report a 1-year OS rate of 76.5%, which is notably better than that reported by Hong et al. [22], but our follow-up is currently limited to one year. The study by Kim et al. [23] did not provide separate OS data for patients undergoing emergency surgery for small bowel tumor complications. We plan to continue following this patient cohort to assess the 5-year OS rate and further clarify long-term outcomes in this population.

There were several limitations in this study. First, this is a retrospective study in a single center and with a small sample size. It included several subtypes and stages of small intestinal NHL which underwent different interventions. In addition, our cohort was not compared with the non-surgical cases of small intestinal NHL.

## Conclusions

In conclusion, our results suggest that emergency management due to complications in patients with primary intestinal lymphoma does not have a negative predictive value on their survival. The prognosis is worse in patients with T-cell primary intestinal lymphomas, which present a higher rate of postoperative complications and recurrence. Prospective and multi-center studies with larger sample sizes are required to confirm these results.

## Data Availability

No datasets were generated or analysed during the current study.

## Declarations

The paper is not based on a previous communication to a society or meeting.
